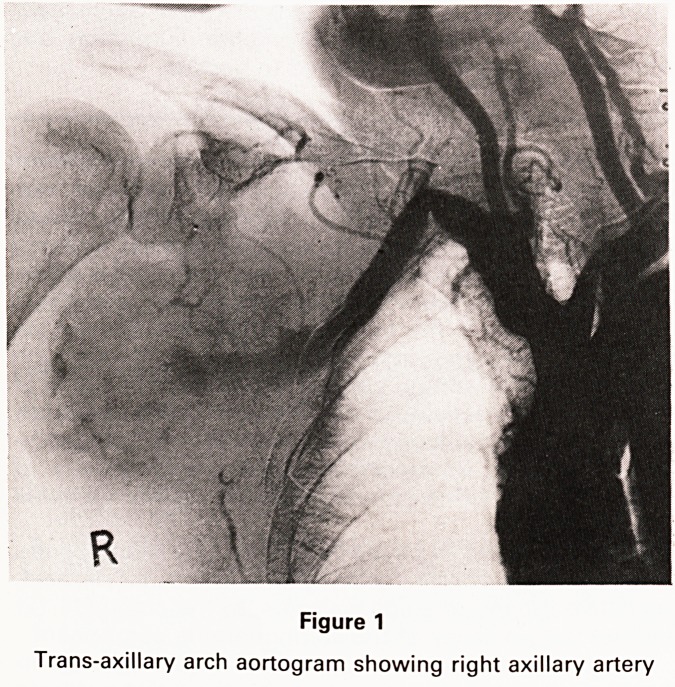# Axillary Aneurysm Presenting as a Brachial Plexus Palsy

**Published:** 1987-08

**Authors:** M. I. Aldoori, W. E. G. Thomas, K. Jeyasingham

**Affiliations:** Senior Surgical Registrar Department of Thoracic Surgery, Frenchay Hospital and Department of Surgery, Bristol Royal Infirmary, Bristol; Senior Surgical Registrar Department of Thoracic Surgery, Frenchay Hospital and Department of Surgery, Bristol Royal Infirmary, Bristol; Consultant Thoracis Surgeon Department of Thoracic Surgery, Frenchay Hospital and Department of Surgery, Bristol Royal Infirmary, Bristol


### Axillary artery aneurysm presenting as a brachial plexus palsy

**M. I. Aldoori FRCS, PhS**
Senior Surgical Registrar
**W. E. G. Thomas FRCS, MS**
Senior Surgical Registrar
**K. Jeyasingham FRCS, FRCSE, ChM**
Consultant Thoracis Surgeon
Department of Thoracic Surgery, Frenchay Hospital
and
Department of Surgery, Bristol Royal Infirmary, Bristol

### INTRODUCTION

The diagnosis of axillary artery aneurysms is often delayed due to the excellent collateral blood supply and the surrounding musculo-skeletal framework which tends to obscure the contents of the axilla.

### CASE REPORT

R. W., an 82 year old man, presented with a painful weakness of the right upper limb following a fall over an armchair. Chest radiography showed "a right apical shadow" and a presumptive diagnosis of Pancoast's tumour was made. Both bronchoscopy and mediastinoscopy were normal. Two weeks later a repeated clinical examination revealed a mass in the right axilla (8 × 8 cm in diameter). The right radial pulse was diminished and there was a discrepancy of 30 mmHg in systolic pressure between the two arms. An arch aortogram was performed and confirmed the presence of a right axillary aneurysm (Figure 1). The apical shadow which was seen on the plain x-ray, was thought to be a knuckle of the brachiocephalic artery. Exploration of the right axillary artery was undertaken approximately five weeks after the onset of the symptoms, and a 7 × 10 cm false axillary aneurysm was found (Figure 1). An 8 mm Teflon graft was used for reconstruction of the vessel. Six months later there was no sign of any neurological improvement in spite of successful revascularization of the limb.

**Figure 1**

Trans-axillary arch aortogram showing right axillary artery

### DISCUSSION

Accurate diagnosis of axillary artery aneurysm is often delayed due to the anatomical position of the aneurysm and the restricted shoulder movements following injury. Distal peripheral pulses may be present despite injury to the axillary artery, due to the excellent collateral circulation (Stromberg and Swedenborg, 1979). Both brachial plexus and axillary blood vessels are invested in a common fascial sheath and therefore brachial plexus palsy may be the earliest and sometimes the only sign of injury to the axillary artery (Donovan and Sharp, 1984). The recovery of any neurological deficit depends upon the speed with which surgical intervention and decompression is carried out (Pairolero et al, 1981), (Donovan and Sharp, 1984). A high index of suspicion, followed by arteriography, should be adopted in order to avoid permanent damage to the brachial plexus.

### REFERENCES

1. DONOVAN, D. L. and SHARP W. V. (1984). Blunt trauma to the axillary artery. *J Vasc Surg* **1**, 681.
2. PAIROLERO, P. C. WALLS, J. T. PAYNE W. S. *et al* (1981). Subclavian-axillary artery aneurysms. *Surgery* **90**, 757.
3. STROMBERG, L. and SWEDENBORG, J. (1979). False aneurysm of the axillary artery following resection of a traumatized humeral head: case report. *J Trauma* **19**, 544.

## Figures and Tables

**Figure 1 f1:**